# A new mutation in the *CAVIN1/PTRF* gene in two siblings with congenital generalized lipodystrophy type 4: case reports and review of the literature

**DOI:** 10.3389/fendo.2023.1212729

**Published:** 2023-07-12

**Authors:** Valentina Mancioppi, Tommaso Daffara, Martina Romanisio, Giovanni Ceccarini, Caterina Pelosini, Ferruccio Santini, Simonetta Bellone, Simona Mellone, Alessio Baricich, Ivana Rabbone, Gianluca Aimaretti, Baris Akinci, Mara Giordano, Flavia Prodam

**Affiliations:** ^1^Division of Pediatrics, Department of Health Sciences, University of Piemonte Orientale, Novara, Italy; ^2^Endocrinology, Department of Translational Medicine, University of Piemonte Orientale, Novara, Italy; ^3^Obesity and Lipodystrophy Center, Endocrinology Unit, University Hospital of Pisa, Pisa, Italy; ^4^Chemistry and Endocrinology Laboratory, Department of Laboratory Medicine, University Hospital of Pisa, Pisa, Italy; ^5^Interdisciplinary Research Center of Autoimmune and Allergic Diseases, University of Piemonte Orientale, Novara, Italy; ^6^Laboratory of Genetics, Struttura Complessa a Direzione Universitaria (SCDU) Biochimica Clinica, Ospedale Maggiore della Carità, Novara, Italy; ^7^Physical Medicine and Rehabilitation, Department of Health Sciences, University of Piemonte Orientale, Novara, Italy; ^8^Division of Endocrinology and Metabolism, Faculty of Medicine, Dokuz Eylul University, Izmir, Türkiye; ^9^Department of Health Sciences, University of Piemonte Orientale, Novara, Italy

**Keywords:** lipodystrophy, CGL4, PTRF, adipose tissue, leptin, muscular dystrophy

## Abstract

Lipodystrophy syndromes are characterized by a progressive metabolic impairment secondary to adipose tissue dysfunction and may have a genetic background. Congenital generalized lipodystrophy type 4 (CGL4) is an extremely rare subtype, caused by mutations in the polymerase I and transcript release factor (*PTRF*) gene. It encodes for a cytoplasmatic protein called caveolae-associated protein 1 (Cavin-1), which, together with caveolin 1, is responsible for the biogenesis of caveolae, being a master regulator of adipose tissue expandability. Cavin-1 is expressed in several tissues, including muscles, thus resulting, when dysfunctional, in a clinical phenotype characterized by the absence of adipose tissue and muscular dystrophy. We herein describe the clinical phenotypes of two siblings in their early childhood, with a phenotype characterized by a generalized reduction of subcutaneous fat, muscular hypertrophy, distinct facial features, myopathy, and atlantoaxial instability. One of the siblings developed paroxysmal supraventricular tachycardia leading to cardiac arrest at 3 months of age. Height and BMI were normal. Blood tests showed elevated CK, a mild increase in liver enzymes and triglycerides levels, and undetectable leptin and adiponectin concentrations. Fasting glucose and HbA1c were normal, while Homeostatic Model Assessment for Insulin Resistance (HOMA-IR) was mildly elevated. Both patients were hyperphagic and had cravings for foods rich in fats and sugars. Genetic testing revealed a novel pathogenic mutation of the *CAVIN1*/*PTRF* gene (NM_012232 exon1:c T21A:p.Y7X) at the homozygous state. The diagnosis of lipodystrophy can be challenging, often requiring a multidisciplinary approach, given the pleiotropic effect, involving several tissues. The coexistence of generalized lack of fat, myopathy with elevated CK levels, arrhythmias, gastrointestinal dysmotility, and skeletal abnormalities should prompt the suspicion for the diagnosis of CGL4, although phenotypic variability may occur.

## Introduction

Lipodystrophies are a heterogeneous group of rare disorders characterized by lack or dysfunction of white adipose tissue (WAT) with perturbation in its mass or distribution (1, 2) and consequent alterations in adipokines levels, mainly leptin and adiponectin (3–5). Lipodystrophies can be classified according to their etiology in genetic or acquired syndromes or based on the extension of the lack of subcutaneous adipose tissue as generalized, partial, or localized (6–10). Manifestations of congenital generalized lipodystrophies (CGLs) appear early, typically at birth or immediately after, and are associated with the development of metabolic complications (11, 12). CGL has four main subtypes, all with an autosomal recessive transmission pattern (13, 14). Their estimated prevalence is approximately 0.23 cases/million and 0.96 cases/million, worldwide and in Europe, respectively (15, 16). Several patients with CGL have been diagnosed among consanguineous individuals, particularly in families from Brazil, Lebanon, Scandinavia, and of African ancestry, probably due to the high rate of founder mutations and endogamy in these ethnic groups (17). To date, about 30 patients with congenital generalized lipodystrophy type 4 (CGL4) have been described, most of them in families of Omani, Japanese, Hispanic, Moroccan, and Turkish origin. In Europe, this phenotype is extremely rare, and only two cases have been reported so far in United Kingdom and Russia (18, 19), with patients reporting consanguinity between their parents or being immigrant families from certain regions where consanguineous relationships are common.

CGL4, first described in 2009 (20), is due to mutations in the polymerase I and transcript release factor (*PTRF*) gene located on chromosome 17q21.2 (21). *PTRF* gene encodes a cytoplasmatic protein called caveolae-associated protein 1 (Cavin-1), which, together with caveolin 1, is responsible for the biogenesis of caveolae and co-localizes in the adipocytes (22, 23), being a master regulator of cell differentiation and adipose tissue expandability (24). Beside adipocytes, Cavin-1 is expressed in several tissues, including muscles (7). Common findings in CGL4 subjects are the following: a progressive loss of adipose tissue during infancy, emaciated face due to the absence of Bichat fat pads, acromegalic features (mainly face, hands, and feet), prominent superficial subcutaneous veins (pseudo-flebomegaly), accelerated growth, umbilical hernia, hirsutism, clitoromegaly and amenorrhea, osteopenia and distal metaphyseal deformation with joint stiffness, pyloric stenosis, atlantoaxial instability, and percussion-induced muscle mounding (2, 8, 25). Patients display lack of the subcutaneous adipose tissue, while the mechanical adipose tissue (primary in the retro-orbital region and in small amounts in the buccal, masticator, para-pharyngeal, infrapatellar, and popliteal regions) and bone marrow fat are preserved (21, 26, 27). Regarding their metabolic profile, subjects may present with hypertriglyceridemia, hepatomegaly and hepatic steatosis, hyperinsulinemia, and insulin resistance, although clear evidence of type 2 diabetes has not been described (1, 11, 19, 28). A peculiar feature of this CGL subtype is the involvement of the skeletal and cardiac muscles. Patients display congenital myopathy with high creatine kinase serum levels, local protracted muscle contractions (also known as “mounding”), and severe arrhythmias usually exercise induced (such as long QT syndrome, catecholaminergic polymorphic ventricular tachycardia) causing an increased risk for sudden cardiac death (18, 29). Hypertrophic cardiomyopathy, with or without hypertension, associated with an ectopic cardiac fat deposition and/or lipotoxicity has been described (30). Some case reports have described nephrosis and transient immunoglobulin A deficiency (20, 31, 32). Patients affected by this disease are expected to be hyperphagic due to the impairment of the feedback between adipocytes and central feeding mechanisms (10). One of the master regulators of this signaling is leptin, an adipokine produced in the WAT, whose levels are directly proportional to body fat mass and are, therefore, extremely low in subjects with CGL (3). Leptin modulates food intake, satiety, and energy balance through its action on hypothalamic neurons (33); therefore, low levels of leptin in lipodystrophy can trigger hyperphagia (34).

We herein describe the clinical phenotypes of two pediatric siblings with CGL4, carrying a novel mutation in exon 1 of the *PTRF* gene in the homozygous state.

## Methods

### Anthropometric measurements and body composition

Birth weight and length were expressed as SD according to WHO growth charts. Height, weight, and waist and hip circumferences (WC and HC, respectively) at our first evaluation were measured by standard procedures, and body mass index (BMI) was calculated. Height, weight, and BMI percentiles and SD were calculated according to the WHO growth charts. Pubertal stages were determined by physical examination, using the criteria of Marshall and Tanner. Skinfold thickness was measured with a Lange caliper (GIMA 27320) at trunk (subscapular) and peripheral (biceps, triceps and calf) sites on the right side of the body. The average of three repeat measurements at each site was calculated. Blood pressure was measured, and systolic (SBP) and diastolic (DBP) values were evaluated according to standard procedure.

Hand-held bioelectric impendence analysis (BIA; TANITA MC-780MA) was used to determine whole-body fat, which was calculated as percentage of body mass. BIA was performed in the male patient, whereas female patients were excluded because age was not comprised in the software formula. BIA percentages were stratified according to the percentile reference value for anthropometric body composition indices in European children from the IDEFICS Study (35).

### Imaging assessment

Abdominal ultrasound (Toshiba, Appler 500) and abdominal MRI (Philips 1.5 Tesla) were performed to evaluate hepatomegaly and hepatic steatosis. The extent of fat loss was evaluated by chest and abdominal MRI. Fibroscan (Echosens 502 F00216) was made to assess liver stiffness, expressed in kPa. For neuromuscular assessment, cervical spine X-ray and spinal column X-ray (Samsung AccE GC85A) were performed to evaluate atlantoaxial instability and scoliosis, respectively. Ventricular hypertrophy and arrhythmia were assessed by two-dimensional echocardiography and electrocardiogram (ECG), respectively. The degree of muscular damage was established through musculoskeletal ultrasounds (Samsung SW2F-45EB). Brain and cervical spinal cord MRI (Philips 3 Tesla) were performed to rule out neurological malformations. The presence of bone lytic lesions was assessed by hand and wrist radiography. Skeletal age was established through hand X-ray, according to the Greulich and Pyle method.

### Biochemistry, hormonal, and immune evaluations

Biochemical and hormonal evaluations were carried out after 12 h of fasting at baseline. They were assessed using standardized methods in the hospital’s chemistry laboratory, particularly the following: serum glucose (mg/dl), insulin (μUI/ml), C-peptide (ng/ml), glycated hemoglobin (mmol/mol), serum total cholesterol (mg/dl), high-density lipoprotein (HDL)-cholesterol (mg/dl), low-density lipoprotein (LDL)-cholesterol (mg/dl), triglycerides (mg/dl), serum aspartate aminotransferase (AST, U/L), alanine aminotransferase (ALT, U/L), gamma glutamyl transferase (γGT, U/L), serum creatinine (mg/dL), serum 25-hydroxy (OH) vitamin D (ng/ml), calcium (mg/dl), phosphorus (mg/dl), parathyroid hormone (PTH, pg/ml), alkaline phosphatase (ALP, U/L), creatine kinase (CK, U/L), insulin-like growth factor 1 (IGF1, ng/ml), growth hormone (GH, ng/ml), serum complement component 3 (C3, mg/dl), complement component 4 (C4 mg/dl), immunoglobulin A (IgA, mg/dl), immunoglobulin G (IgG, mg/dl), and immunoglobulin M (IgM, mg/dl).

Serum leptin (mcg/L) and adiponectin (mcg/ml) were measured by CLIA from Mediagnost, Reutlingen, Germany.

### Genetic analysis

The DNA was extracted from 200 µl of whole blood (anti-coagulated with EDTA) using ReliaPrep™ Blood gDNA Miniprep System (Promega, Fitchburg, WI, USA). For clinical exome sequencing panel, the Agilent SureSelect Custom Constitutional Panel 17 Mb (Agilent, Santa Clara, CA, USA) was used, which includes 5,227 clinically relevant genes. Target enrichment was performed for all designs using the Agilent SureSelect^QXT^ NGS target enrichment kit for Illumina multiplexed sequencing (Agilent Technologies). Sequencing was performed using Illumina MiSeq (Illumina, UK) and MiSeq v3 300 Cycle Reagent Kits. Sequence alignment, annotation, categorization, and variant calling were performed using SureCall v3.5 software (Agilent), and Variant Call Format (VCF) files were annotated with wANNOVAR (http://wannovar.wglab.org) software. The filtering of the variants was based on their frequency in the public databases: 1000 Genome project (http://browser.1000genomes.org), EXAC (http://exac.broadinstitute.org), dbSNP (http://www.ncbi.nlm.nih.gov/projects/SNP/), and gnomAD (https://gnomad.broadinstitute.org/). All the variants that were not present in public databases and variants with a minor allele frequency (MAF) <1% were considered as potentially pathogenic.

The variant (NM_012232 exon1:c T21A:p.Y7X) identified by clinical-exome sequencing was validated by amplifying *PTRF* exon 1 with primers 5′-AGCCAATCAGCGATCAGACT-3′ (forward) and 5′-GATTTTGTCCAGGAGGCTCA-3′ followed by Sanger sequencing.

### Informed consent

Patients and parents gave their informed consent for genetic studies and publication of their clinical details and images.

## Results

### Patient 1

The oldest of the two siblings was a male Caucasian, born full-term after an uncomplicated pregnancy and delivery in Romania. At birth, his weight was 3.2 kg (−0.69 SD), and length was 50 cm (−0.40 SD). Parents reported not being consanguineous, but they came from the same small Moldavian town. They referred that a male cousin distantly related to both of them, with a phenotype resembling patient 1, died at the age of 40 years from cardiac complications. At 40 days of age, patient 1 was admitted to the hospital for forceful vomiting and underwent surgery for pyloric stenosis. He also suffered from left inguinal hernia, gastroesophageal reflux disease, and lower airway infections, which often required hospitalization (a total of nine admissions for pneumonia in the first 4 years of life occurred). Parents referred that the subcutaneous fat loss became evident during the first months of life, especially in the face and limbs. Motor milestones and mental development were normal. He started walking at 10 months and speaking approximately 15 months of age. His height was always within the target height (TH), weight <3° percentile, and BMI between 3° and 10° percentile. After weaning, parents noticed that he was hyperphagic, mainly for food rich in fats and sugars. The patient’s walking rapidly deteriorated, and at the age of 2 years, he was only able to walk on his toes. Biochemical assessments revealed elevated serum CK levels, IgA deficiency, normal IgG levels, and mildly increased IgM levels. Total cholesterol and fasting glucose levels were within the normal range. Mild hypertriglyceridemia and impaired liver function were detected (**Supplementary Table S1**). The echocardiogram showed a diastolic dysfunction of the right ventricle, ostium secundum atrial septal defect, tricuspidal reflux (I–II grade), and patent ductus arteriosus (PDA). The abdominal ultrasonography detected hepatomegaly with initial steatosis. Due to the persistently elevated CK levels, he underwent muscle biopsy that showed dystrophic changes (variation in fiber size, necrotic fibers, and fibers with atypical regenerating form). Electromyography and nerve conduction studies were normal.

At our first evaluation, the patient was 5.2 years old, his height was 113.4 cm (58° percentile, +0.21SD) within the target height (TH); weight (19.5 kg, 41° percentile, −0.24 SD) and BMI (15.16 kg/m^2^, 32° percentile, −0.47 SD) were normal. Waist and hip circumferences were 54 and 55 cm, respectively. Skinfold thickness showed a reduction in subcutaneous fat tissue in the upper and lower limbs (<1° percentile) (**Table 1**). His body fat percentage at BIA was 11%.

**Table 1:** Clinical features of patients.

	Patient 1	Patient 2
**Sex**	M	F
**Age (years)**	5.2	2.2
**Height (cm/SD)**	113.4/+0.21	89.1/+0.0
**Weight (cm/SD)**	19.5/−0.24	11.8/−0.65
**BMI (kg/m^2^/SD)**	15.1/−0.47	14.8/−0.76
**Growth velocity (cm/year/SD)**	6.26/−0.01	11.14/1.94
**Pubertal stage**	Prepuberal	Prepuberal
**Hypertension**	No	No
**WC (cm/percentile)**	54/75°	48/75°
**HC (cm)**	55	45
Whole body fat (%/percentile)*	11/3°–10°	–
Skinfold thickness		
**Tricep dx (mm/percentile)**	3.2/<1°	4.0/<1°
**Tricep sx (mm/percentile)**	3.9/<1°	3.9/<1°
**Subscapular dx (mm/percentile)**	4.2/3°–10°	5.2/10°–25°
**Subscapular sx (mm/percentile)**	5.0/25°–50°	5.2/10°–25°
**Calf dx (mm)**	3.0	4.1
**Calf sx (mm)**	2.9	4.0

*Body fat measured by BIA. BMI, body mass index; HC, hip circumference; NA, not available; WC, waist circumference.

**Figure 1:**
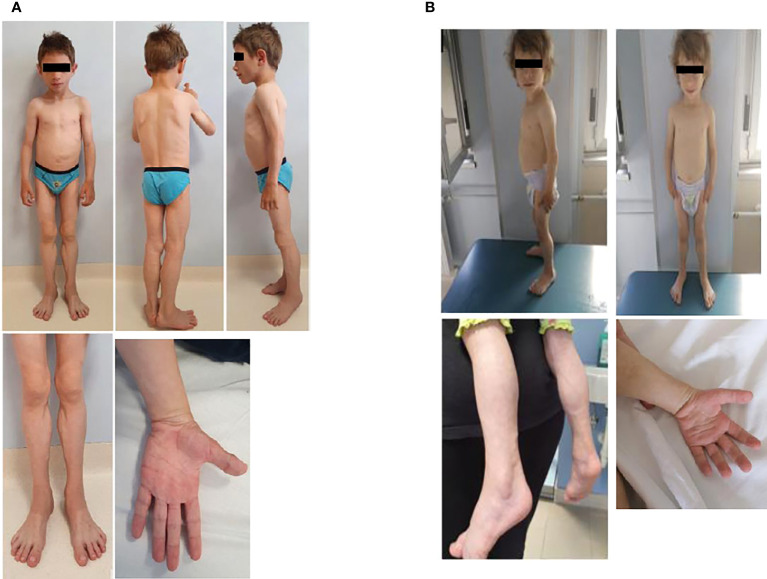
Anterior and side view of the male **(A)** and the female **(B)**. Pictures show the generalized loss of subcutaneous adipose tissue leading to prominence of muscular hypertrophy (more evident in the lower limbs) and prominent veins. The male patient has also progeroid facial features and an obliged posture on his toes, while the female has an acromegaloid appearance.

Physical examination was characterized by generalized lack of subcutaneous fat, with preservation in the palms and soles. Acromegaloid appearance, muscular hypertrophy (more evident in the lower limbs), prominent veins, broad nasal bridge, mandibular hypoplasia, and hypertelorism were detected (**Figure 1**). Liver and spleen were palpable 1 cm below the costal margin, and he had umbilical prominence; no acanthosis nigricans was visible. His genitalia were prepubertal, and testes were approximately 2 ml bilaterally. He could only walk on his toes due to the shortening of the Achilles tendon, keeping a posture of anterior trunk flexion to compensate. Deep tendon reflexes were present. He did not demonstrate percussion-induced muscle mounding or the rippling phenomenon. Mental development was normal.

Biochemical blood tests (**Table 2**) showed mild hypertriglyceridemia, normal cholesterol levels, IgA deficiency, mildly increased IgM, impaired liver function, normal thyroid, parathyroid, and adrenal functions. He had a normal fasting glucose profile but had initial signs of insulin resistance. CK levels were very high. Renal function was normal, with no proteinuria. IGF-1 values were in the upper end of the normal range, and GH pulsatility was normal. Serum leptin and adiponectin levels were undetectable.

**Table 2:** Biochemical evaluation of the two siblings.

	Patient 1	Patient 2	Normal Value
**Sex**	M	F	–
**Glucose (fasting) (mg/dl)**	83	73	70–100
**HbA1c (%)**	5.2	4.7	4.2–6.2
**Insulin (fasting) (μUI/ml)**	7.0	5.1	6.0–27.0
**HOMA-IR**	1.43	0.92	<1.03*
**C-peptide (ng/ml)**	0.96	0.72	0.80–4.20
**Total-C (mg/dl)/percentile**	143/<50°	145	<180
**HDL-C (mg/dl)/percentile**	46/10°–25°	33	>60
**LDL-C (mg/dl)/percentile**	80/<50°	84	<110
**TG (mg/dl)/percentile**	85/95°	139	<75
**AST (U/L)**	37	63	0–40
**ALT (U/L)**	53	62	0–40
**γGT (U/L)**	8	11	0–50
**CPK (U/L)**	898	1348	38–174
**25OH Vitamin D (ng/ml)**	25.5	19.5	30–100
**PTH (pg/ml)**	19.8	25.7	6.5–39.0
**Calcium (mg/dl)**	9.9	9.7	8.6–10.0
**Phosphorus (mg/dl)**	6.0	6.5	2.7–4.5
**ALP (U/L)**	390	520	140–400
**IGF-1 (ng/ml)**	153.5	185.9	54.9–206.4** 33.5–171.8***
**Leptin (μg/L)**	<0.2	< 0.2	0.65–13.4
**Adiponectin (μg/ml)**	<0.2	0.3	1.04–13.58
**C3 (mg/dl)**	94	81	90–180
**C4 (mg/dl)**	17	12	10–40
**IgA (mg/dl)**	67	< 20	55–152** 19–235** *
**IgM (mg/dl)**	157	79	22–100** 28–113***
**IgG (mg/dl)**	633	388	569–1,597** 45–1,236***

*HOMA-IR value <90° percentile.

**Reference range corrected for the male age.

***Reference range corrected for the female age.

25 OH Vitamin D, 25-hydroxy (OH) vitamin D; ALP, alkaline phosphatase; ALT, alanine aminotransferase; AST, aspartate aminotransferase; C3, complement component 3; C4, complement component 4; CPK, creatine phosphokinase; FA, alkaline phosphatase; γGT, gamma glutamyl transferase; HbA1c, hemoglobin A1c; HDL-C, high-density lipoprotein cholesterol; IgA, immunoglobulin A; IgG, immunoglobulin G; IgM, immunoglobulin M; IGF-1, insulin-like growth factor 1; LDL-C, low-density lipoprotein cholesterol; total-C, total cholesterol; PTH, parathyroid hormone; TG, triglycerides.

Cervical X-ray detected atlantoaxial instability with predental space of 3.5 mm in neutral position, which increased to 4 mm during flexion (**Supplementary Figure S1A**); the spinal column X-ray revealed mild scoliosis (**Supplementary Figure S1B**). Brain and cervical spinal cord MRI ruled out neurological malformations. He did not report neck pain, and no signs of myelopathy could be observed.

Resting ECG was normal with a regular QT interval, but no exercise test was undertaken on the boy, due to his young age, to search for exercise-induced arrythmias. Echocardiography showed mild mitral regurgitation and patent foramen ovale (PFO), without signs of ventricular hypertrophy.

**Figure 2:**
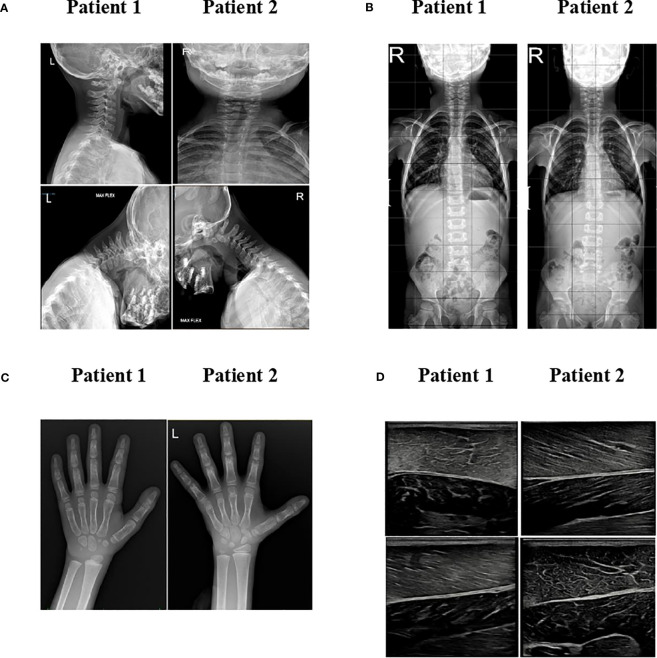
MRI imaging. T1-weighted MR imaging of patients 1 and 2 in comparison with a normal subject at different levels. Adipose tissue has a high signal intensity (brightness) on MRI T1-weighted images. When compared to normal subjects, the two patients have marked loss of subcutaneous fat, except for retro-orbital and bone marrow fat.

The hand and wrist radiography showed a bone age more than 2 years advanced, no signs of cystic lesions, but a reduction in the calcium content with alteration of the trabecular bone mass (**Supplementary Figure S1C**). Abdominal ultrasound and MRI displayed hepatomegaly without hepatic steatosis. No liver stiffness was detected at fibroscan (E, 4.9 kPa). Generalized loss of adipose tissue was evident at T1-weighted head, chest, and abdominal body MRI (**Figure 2**). Musculoskeletal ultrasound revealed dystrophic changes in muscle fibers (**Supplementary Figure S1D**).

### Patient 2

The sister of patient 1 was born full-term after an uncomplicated pregnancy and delivery in Romania. At birth, her weight was 3.7 kg (+0.85 SD), and the length was 49 cm (−0.75 SD). At 3 months of age, the patient suffered a cardiac arrest resolved after cardiopulmonary resuscitation. The cardiac assessment revealed a paroxysmal supraventricular tachycardia (PSVT). The child was placed on therapy with beta-blocker and digoxin until she was 1 year old and did not experience further episodes of arrhythmia ever since. Like her brother, a lack of subcutaneous fat was noticed during the first months of life, especially in the face and limbs. She showed normal motor milestones and mental development: she started speaking approximately 15 months of age and walking at 12 months, preferably on her toes. She also presented hyperphagia and preference for food rich in fats and sugars. Her height was always within the target height, weight was 10°–25° percentile, and BMI was between 10° and 25° percentile. Biochemical assessments are shown in **Supplementary Table S1**. The abdominal ultrasonography showed no signs of hepatomegaly or steatosis.

At our first evaluation, she was 2.2 years old, her height was within the TH (89.1 cm, 50° percentile, +0.0 SD), and weight (11.8 kg, 26° percentile, −0.65 SD) and BMI (14.8 kg/m^2^, 22° percentile, −0.76 SD) were normal. Waist and hip circumferences were 48 and 45 cm, respectively. Skinfold thickness showed a reduction in subcutaneous fat in the upper and lower limb (<1° percentile) (**Table 1**). BIA was not performed due to her young age. Physical examination revealed near total of lack of fat with preservation of fat in the palms and soles. She also displayed muscular hypertrophy (more evident in the lower limbs) and prominent veins, typical facial features, a triangular face, broad nasal bridge, mandibular hypoplasia, hypertelorism, periorbital edema, and cutis marmorata. No acanthosis nigricans was visible (**Figure 1**). Her genitalia were prepubertal with clitoromegaly. She preferred walking on her toes but was able to walk normally, keeping a posture of minimal anterior trunk flexion. Deep tendon reflexes were normal, and percussion-induced muscle mounding or the rippling phenomenon was absent. Mental development was normal.

Biochemical blood tests resembled those of the brother (**Table 2**). Her fasting glucose was normal with no increase in Homeostatic Model Assessment for Insulin Resistance (HOMA-IR) yet. Her CK levels were increased. IGF-1 concentration was slightly increased, but GH pulsatility was normal. Serum leptin and adiponectin levels were undetectable.

Cervical X-ray detected atlantoaxial instability with predental space of 2.5 mm in neutral position, which increased to 4 mm during flexion (**Supplementary Figure S1A**); the spinal column X-ray revealed mild scoliosis (**Supplementary Figure S1B**). Like her brother, she did not have neck pain, and there were no signs of myelopathy. Brain and cervical spinal cord MRI ruled out neurological malformations.

Echocardiography did not show signs of ventricular hypertrophy. The hand and wrist radiography showed a bone age 1 year advanced, with no signs of cystic lesions, but like her brother, a reduction in the calcium content with alteration of the trabecular bone mass was observed (**Supplementary Figure S1C**).

Abdominal ultrasound and MRI displayed hepatomegaly without hepatic steatosis. Elastography showed increased liver stiffness (5.1 kPA). T1-weighted body MRI was very similar to patient 1, showing generalized loss of adipose tissue (**Figure 2**). Musculoskeletal ultrasound revealed dystrophic changes in muscle fibers with adipocytes infiltrations (**Supplementary Figure S1D**).

### Genetic analysis

Clinical exome sequencing performed in the male patient revealed the presence of a novel pathogenic variation at the homozygous state in exon 1 of the *CAVIN1/PTRF* gene (NM_012232.6), namely, c.21T>A that converts the amino acid tyrosine at position 7 of the protein in a stop codon (p.Tyr7Ter), thus causing the premature truncation of the protein. The variant, validated by Sanger sequencing, was inherited from the parents who were both heterozygous for this mutation (**Supplementary Figure S2**).

Genetic analysis in the sister confirmed the presence of the same pathogenic variation in exon 1 of the PTRF gene (NM_012232.6 c.21T>A;p.Tyr7Ter).

## Discussion

This study describes the phenotypic and clinical characteristics of two pediatric siblings affected by CGL type 4 due to a novel homozygous mutation in the *PTRF/CAVIN 1* gene.

CGL4 is a recently identified subtype of CGL (CGL4, MIM 613327) characterized by distinctive clinical features such as myopathy, gastrointestinal abnormalities, cardiac arrythmias, and cervical spine instability (36). Initially, CGL4 was phenotypically described in two Mexican American siblings as a novel subtype of CGL (37). Afterwards, mutations in the polymerase I and transcript release factor (PTRF) gene, encoding a cytoplasmatic protein called caveolae-associated protein 1 (Cavin-1), essential for caveolae formation, were described as the etiology (20). To date, 15 mutations (involving 30 patients) of this gene have been described in HGMD (http://www.hgmd.cf.ac.uk/ac/index.php) and diagnosed in several countries (Japan, Saudi Arabia, Oman, Egyptian, Turkey, the United Kingdom, Germany, Morocco, and Mexico). Previously, five missense/nonsense mutations, two splice sites, five small deletions, and three small insertions were discovered in the *CAVIN1/PTRF* gene (18–20, 28, 30, 36–46). We herein describe a novel homozygous mutation (c.21T>A; p.Tyr7Ter) that causes a change in the reading frame to stop codon, resulting in a premature protein truncation with a likely loss of function effect. Interestingly, the majority of patients with CGL4 reported so far had null mutations in the *CAVIN1/PTRF* gene, exhibiting classical characteristics of the disease similar to our cases. Although limited, milder phenotypes have been described in rare cases, suggesting phenotypic variability in CGL4. A single base deletion (c.947delA) resulting in a frameshift, depletion of the normal stop codon, and extension of the 3′ untranslated region (UTR) reading frame of the CAVIN1/PTRF gene, producing a predicted protein of higher molecular weight, was described in a Moroccan child who was reported to have a subtle phenotype of CGL4 (41). This prompted the question of whether less severe mutations, such as missense mutations, could be associated with CGL4 of less severe phenotypes. Mild metabolic disorders in heterozygous carriers were reported, suggesting that these individuals may be at risk for mild metabolic disturbances even though they do not exhibit overt lipodystrophy or myopathy (18, 25).

Both of our patients showed early metabolic complications such as hypertriglyceridemia, low HDL cholesterol levels, hepatomegaly, and hepatic steatosis with elevated levels of liver enzymes. A slight increase in HOMA-IR was observed only in one patient. The metabolic phenotype was similar to other previously described CGL4 cases (19, 38, 39, 45, 47). Dyslipidemia is a common finding among these patients, even in the first years of life, whereas diabetes tends to develop during puberty, but subjects can express elevated fasting insulin levels and mild insulin resistance at a younger age. In line with previous reports, none of our siblings had proteinuria or impaired renal function (31, 34). This could be explained by the younger age of our cases compared to the expected age of the onset of this comorbidity (31, 34). The relationship between generalized lipodystrophy and metabolic derangements is well known. Looking specifically at CGL4, PTRF-CAVIN protein is a master regulator in the process of caveolae stabilization (22, 23). The lack of caveolae can affect the regulation of lipolysis, fatty acid flux, triglyceride synthesis, and alteration of insulin signaling in adipocytes, as caveolae are known to concentrate insulin receptors along their margins (14, 39). Caveolae function is extremely complex, including the compartmentalization of multiple signaling pathways not related to the metabolism. A recent case report has shown how the immune system of a subject with CGL4 did not mount a normal response to a single dose of lipid nanoparticle encapsulated mRNA anti-SARS-CoV-2 vaccine but responded to a single dose of the Adenovirus-based vaccine (48). The authors hypothesized that the poor patient’s immune response could be related to the reduced import of the vaccine due to the lack of caveolin, since the lipid nanoparticle encapsulated mRNA is internalized by caveolae-mediated endocytosis.

Patients with CGL4 have been described to present a milder metabolic profile compared to the other subtypes of CGL (20, 28, 36, 38), but several biases may intervene to confound the clinical picture, including the age at diagnosis, the dietary regimen, and lifestyle of various affected subjects. In the past, authors hypothesized that the missense mutations frequently observed in patients with CGL4 could cause a milder metabolic phenotype (28). Another possible explanation could be related to a relatively later loss of secretory function of adipocytes, mainly leptin and adiponectin than other CGLs (10). However, both our patients showed very low/undetectable levels of leptin and adiponectin at very young ages, similarly to most children with CGL4 described by others (31–33, 40). In addition, patients with CGL1 or CGL2, the subtype associated with the most severe metabolic phenotypes, had not only low leptin and adiponectin levels but also very low circulating free leptin and high-soluble leptin receptor levels (49). Since the circulating free leptin isoform is the biologically active subtype, this form could determine the clinically divergent metabolic phenotype. We could therefore speculate that patients with CGL4 have higher circulating free leptin levels and lower protein-bound forms than other CGLs, causing the milder phenotype. Further studies on these patients are necessary to confirm this hypothesis. Other possible explanations for the milder phenotype could be related to the younger age of our patients, preventing the natural disease progression, which typically occurs after early childhood, or related to the gastrointestinal disease potentially limiting food intake or the hypertrophic muscles potentially consuming a portion of surplus energy.

Body composition studies highlighted a reduction in whole subcutaneous adipose tissue, especially over the extremities. MRI scans revealed the characteristic pattern of fat distribution, with generalized loss of SAT but preserved mechanical fat in the retroorbital and periarticular regions, over the palms and soles and in the bone marrow fat. These results were similar to those demonstrated in several studies regarding CGL4 (19, 29, 37, 38), except for the presence of adipose tissue in the palms and soles that was not described in older patients (38). We could hypothesize that the adipose tissue loss in the hands and feet could manifest later in life, typically when the children are in puberty, contrary to the metabolic adipose tissue loss that is already evident from the first months of life.

However, despite the decreased body fat, children had normal height and growth rate was accelerated, likely a result of the relatively high IGF-1 values detected in the patients despite normal GH pulsatility. Since caveolae constitute one pathway for the internalization of GH *in vitro* (18, 46), the elevated growth velocity and IGF-1 levels, observed in our children with CGL4, could be associated with the reducing caveolae formations, typical of this subtype of lipodystrophy, since GH pulsatility was normal.

The role of leptin as a regulator of bone mass is controversial (50, 51). However, children and adults affected by CGL had no alterations in bone metabolism markers, despite leptin deficiency (52, 53). In our patients, hand and wrist X-ray of our siblings showed a reduction in the calcium content with trabecular bone mass alteration, while baseline serum markers of bone metabolism were within the normal range, except for hypovitaminosis D, and no past fractures were reported. The low calcium content could be related to hypovitaminosis D and the increased growth rate at the time of diagnosis. The follow-up will help clarify bone metabolism during growth.

Apart from the bone, CGL4 importantly affects skeletal, cardiac, and gastrointestinal muscles (21, 23). Bioptic, electron microscopy, and US studies have shown chronic dystrophic changes, including marked variation in muscle fiber size, internal nucleation, a few necrotic and regenerating fibers, increased interstitial fibrosis with reduced caveolae formation, and accumulation of lipid droplets (20, 29, 39–41). Although the exact pathogenesis of myopathy remains unclear, it is common thought that the lack of caveolae has a crucial role in the pathogenesis of the muscle phenotype (30). In our patients, muscle abnormality was denounced during the first years of life, as demonstrated by rapidly deteriorating walking ability and extremely elevated serum CK levels. The muscle biopsy performed on the boy showed variation in fiber size, necrotic fibers, and atypical regenerating forms. At ultrasound, muscles appeared hyperechogenic due to replacement of the hypoechogenic muscle bundles with adipocytes. Tight Achilles tendon is another hallmark of CGL4 caused by hypertrophic calves. Patients are not able to walk on their feet but are forced to stay on their toes, which dramatically increases forefoot pressures (29). In agreement with the literature, both of our patients suffered from Achilles tendon retraction from the first years of life, with the boy presenting a more severe phenotype. Other interesting features in both children were scoliosis and atlantoaxial dislocation, whereas we did not observe percussion-induced muscle mounding, a peculiar feature of CGL4 and of muscular dystrophy due to mutations in the CAV3 gene (19, 29, 40). In the latter disease, preservation of sarcolemmal caveolin-3 staining pattern was observed in young patients compared to older subjects who showed a complete deficiency of caveolin-3, associated with percussion-induced muscle mounding (20, 40, 41). We may hypothesize that in patients with CGL4, secondary reduction in caveolin-3 might arise with age and/or disease progression, and only when deficiency of both proteins (caveolin-3 and cavin-1) develops that muscle mounding can be observed.

Gastrointestinal tract disorders have been described in СGL4 to underline the role of cavin-1 in muscle functionality (18, 20, 28, 38, 42, 45). Massive smooth muscle hypertrophy and thick muscularis mucosa have been shown in CGL4 patients (18, 30). Our male patient had a history of pyloric stenosis, while his sister did not show signs of gastrointestinal involvement, suggesting a phenotypic variability in the presence of the same mutation. The ultrasound study of the gut was normal, but tight dysmotility could be hypothesized since both children, mainly the male one, frequently suffer from abdominal pain not responsive to classic gastrointestinal care advice.

Cardiac arrhythmia have been reported in many CGL4 patients, with an increased risk of sudden death, likely secondary to ventricular arrhythmia also during adolescence (18, 20, 28, 30, 38, 41, 45, 54). The underlying mechanism of paroxysmal ventricular tachycardia (PVT) in patients with CGL4 is unclear. Autoptic studies showed adipocytes interdigitating muscle fibers and mild fibro-fatty infiltration close to the coronary sinus area and atrioventricular node (30). Therefore, the novel cardiac pathology could promote the development of progressive cardiomyopathy, with left ventricular hypertrophy and interstitial/perivascular fibrosis around cardiomyocytes with an alteration of membrane polarization. Mutations in *CAV3* gene, also involved in the caveolae formation, induce an increase in late sodium current by affecting the sodium channels located in the caveolae (28); we could hypothesize that *PTRF/CAVIN1* mutations in patients affect the sodium channel or the other cardiac channels. In resting conditions, no arrhythmia episodes are described in patients with CGL4. However, during exercise when the heart is subjected to a more significant physical effort, due to the possible mechanism previously described, arrhythmias could develop. Since cardiac arrhythmia is a severe and potentially life-threatening condition, occurring frequently after emotions or exercise, patients with CGL4 should be closely monitored by ECG and, if necessary, fitted with an implanted pacemaker and cardioverter defibrillator (ICD) device. The girl, but not the boy, suffered from paroxysmal supraventricular tachycardia (PSVT) at 3 months of age, causing a cardiac arrest, suggesting again a significant degree of phenotypic variability. After cardiopulmonary resuscitation, the child started therapy with beta-blocker and digoxin until she was 1 year old. No other episodes have been described since then. Echocardiography did not show signs of ventricular hypertrophy in both children. However, they are under tight control, and physical activity has been scheduled after cardiologist and physiatrist indications and monitoring.

## Conclusion

We have herein described the phenotype of two pediatric siblings affected by CGL4 due to a novel homozygous mutation of the *CAVIN1/PTRF* gene. The coexistence of generalized lack of fat, myopathy with elevated CK levels, arrhythmias, gastrointestinal dysmotility, and skeletal abnormalities should prompt a diagnosis of CGL4, although phenotypic variability could be present.

CGL4 in Europe is extremely rare, and only two other cases have been described so far, while the majority of patients come from families of Omani, Japanese, Hispanic, Moroccan, and Turkish origin.

Most patients from these countries, described so far, have identical point mutations, suggesting that they have all the same origin, while in Europe, the genetic etiology of CGL4 previously reported is more complex due to a variability in patients with different ethnic backgrounds, and the presence of immigrant families form different parts of the world. The diagnosis of patients with CGL4 with a heterogeneous molecular etiology is fundamental to detail variable phenotypic expressions, as was observed in our patients.

## Data availability statement

The datasets presented in this article are not readily available because of ethical and privacy restrictions. Requests to access the datasets should be directed to the corresponding author/s.

## Ethics statement

The studies involving human participants were reviewed and approved by the Ethics Committee of Maggiore Hospital Novara (protocol code 69/19 and date of approval 2019). Written informed consent to participate in this study was provided by the participants' legal guardian/next of kin. Written informed consent was obtained from the participants/patients' legal guardian/next of keen for the publication of this case report.

## Author contributions

VM, TD, MR, and FP collected the anamnestic and the biochemical data for the patients. VM, AB, and TD performed the literature search, reviewed, and extracted data from the papers. MG, SM, and CP performed the genetic and hormones analysis. VM and SB performed the figures and table designing. VM, FP, SM, and MG wrote the manuscript. FP, BA, GA, GC, FS, and IR verified the analytical methods and supervised the manuscript drafting. All authors discussed the results and contributed to the final manuscript. All authors contributed to the article and approved the submitted version.
