# Supplementary material for: A new mutation in the CAVIN1/PTRF gene in two siblings with congenital generalized lipodystrophy type 4: case reports and review of the literature
Source: Front Endocrinol (Lausanne). 2023 Jul 12;14:1212729. doi: 10.3389/fendo.2023.1212729 (PMC10369054; doi:10.3389/fendo.2023.1212729)
Supplement: Supplementary file 1 [file Image_1.pdf]

Figure S1. Radiological images of the two children

A. Patient 1 Patient 2

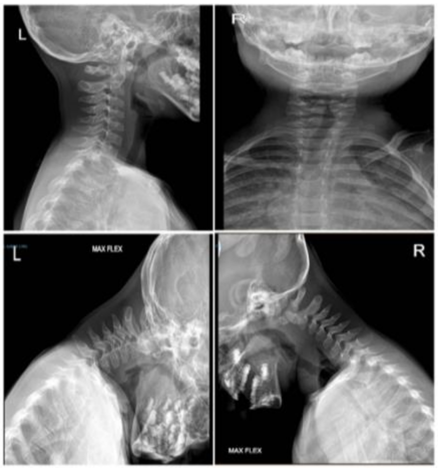

B. Patient 1 Patient 2

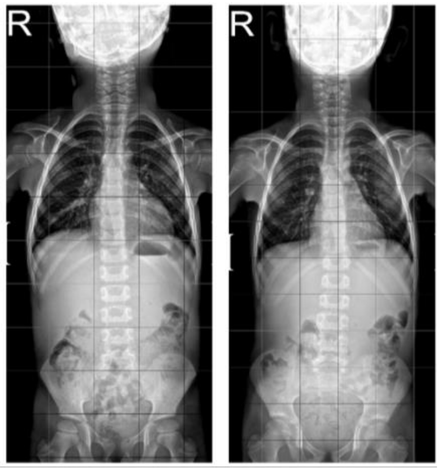

C. Patient 1 Patient 2

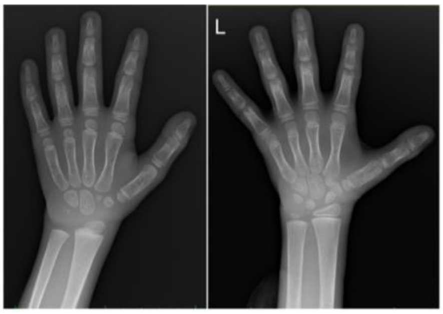

D. Patient 1 Patient 2

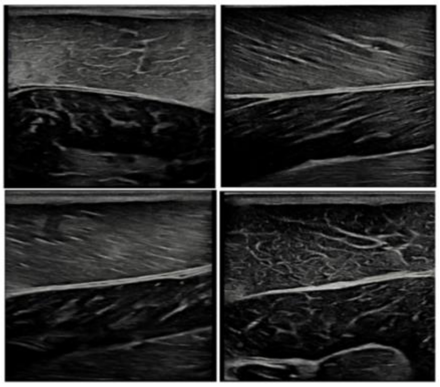

(A). Latero cervical- frontal spine X-rays of Patient 1 and Patient 2 in neutral and flexion position of the neck. Patient 1 X-ray shows a predental space of 3.5 mm in neutral position and increased to 4 mm during flexion. Patient 2 X-ray shows a predental space of 2.5 mm in neutral position and increased to 4 mm during flexion. (**B).** Frontal spine X-ray shows mild scoliosis in both patients. (**C).** Hand and wrist radiography of the two siblings showing a reduction of the calcium content with trabecular bone mass alteration, especially in the metacarpal bones. (**D).** The muscle appears hyperechogenic at the ultrasound due to the replacement, muscle tissue, of the hypoechogenic muscle bundles with the hyperechogenic adipocytes.
