# Supplementary material for: A new mutation in the CAVIN1/PTRF gene in two siblings with congenital generalized lipodystrophy type 4: case reports and review of the literature
Source: Front Endocrinol (Lausanne). 2023 Jul 12;14:1212729. doi: 10.3389/fendo.2023.1212729 (PMC10369054; doi:10.3389/fendo.2023.1212729)
Supplement: Supplementary file 2 [file Image_2.pdf]

Figure S2. Genetic analysis

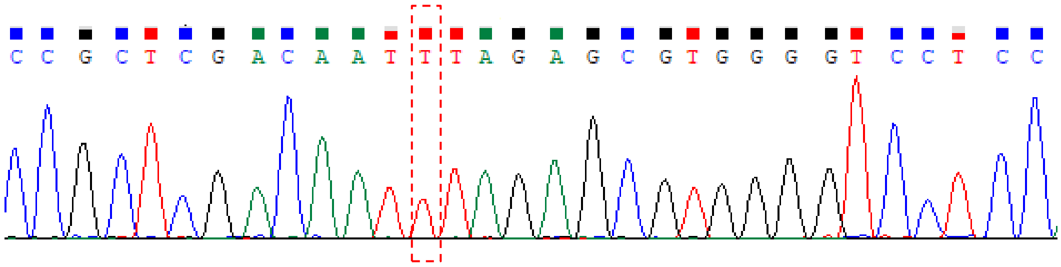

Sanger sequencing chromatograms reporting the homozygous *CAVIN1/PTRF* mutation (NM_012232 eson1:c T21A:p. Y7X) identified in the patients.
