# Supplementary material for: A new mutation in the CAVIN1/PTRF gene in two siblings with congenital generalized lipodystrophy type 4: case reports and review of the literature
Source: Front Endocrinol (Lausanne). 2023 Jul 12;14:1212729. doi: 10.3389/fendo.2023.1212729 (PMC10369054; doi:10.3389/fendo.2023.1212729)
Supplement: Supplementary file 3 [file Table_1.pdf]

Table S1 Biochemical evaluation performed before hospital admitting

	Patient 1	Patient 2	Normal Value
**Sex**	M	F	-
**Total-C (mg/dl)**	173	169	< 180
**TG (mg/dl)**	100	154	< 75
**ALT (U/L)**	58	56	5 - 38
**AST (U/L)**	49	40	5 - 38
**Glucose (mg/dl)**	80	63	< 100
**CK (U/L)**	1120	1290	24 - 225
**IgA (mg/dl)**	55	8	68 – 152 *<br>19 – 119 **
**IgG (mg/dl)**	662	436	569 – 1597 *<br>256 – 1393 **
**IgM (mg/dl)**	141	240	2 – 100 *<br>14 – 114 **

* Reference range corrected for the male age. ** Reference range corrected for the female age. Abbreviations: ALT, alanine aminotransferase; AST, aspartate aminotransferase; CPK, creatine phosphokinase; IgA, immunoglobulin A; IgG, immunoglobulin G; IgM, immunoglobulin M; total-C, total cholesterol; TG, triglycerides.
